# A reversible rat model of hyposmia affects respiration-linked brain oscillations and behavior

**DOI:** 10.1016/j.isci.2026.115760

**Published:** 2026-04-15

**Authors:** Wiktoria Podolecka, Aleksandra Bramorska, Mark Jeremy Hunt

**Affiliations:** 1 Nencki Institute of Experimental Biology of Polish Academy of Sciences, 3 Pasteur Street, Warsaw 02-093, Poland

**Keywords:** neuroscience, behavioral neuroscience, sensory neuroscience, cognitive neuroscience

## Abstract

Rhythmic breathing coordinates neural activity across olfactory and limbic circuits by coupling nasal airflow to oscillations in the olfactory bulb (OB) and downstream networks. To test how reduced peripheral olfactory input reshapes this respiration-linked coordination, we developed a reversible hyposmia model in rats using intranasal gadolinium chloride. This manipulation impaired hidden cookie performance for around 1 week and was accompanied by nasal epithelial damage. Hyposmia robustly decreased OB respiration-linked (1–10 Hz) and gamma (30–90 Hz) power, with smaller reductions in prefrontal cortex and ventral striatum. In the OB, oscillatory changes were confined to wakefulness, while slow-wave sleep activity was largely preserved, consistent with intact top-down drive during sleep. Additionally, hyposmia increased anxiety-like behavior and produced minor changes in recognition memory without altering locomotion or hedonic drive. Together, these findings connect peripheral nasal function to state-dependent brain synchrony and affect-related behavior.

## Highlights

• Intranasal gadolinium induces a short-lasting, reversible hyposmia in rats
• Hyposmia reduces respiration-linked and gamma oscillations in the olfactory bulb
• Wake-related, but not sleep-related, olfactory bulb rhythms depend on nasal input
• Transient hyposmia increases anxiety-like behavior and impairs recognition memory

## Graphical abstract

**Figure:**
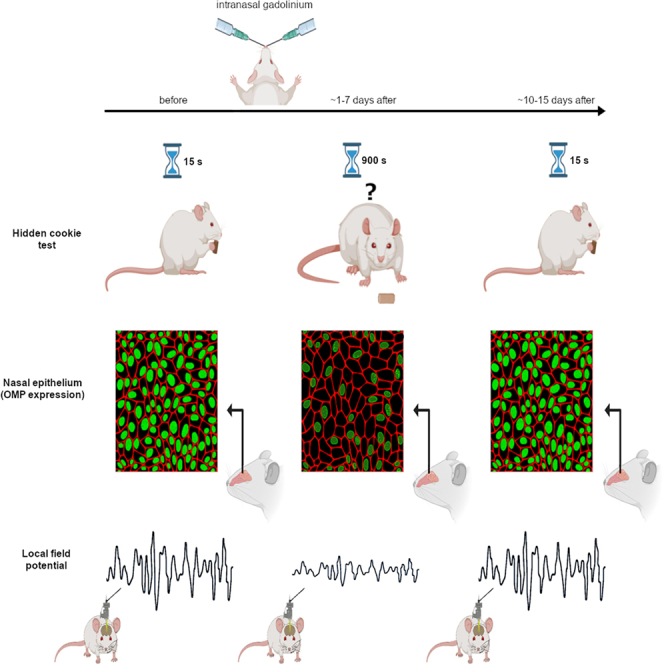

## Introduction

Nasal airflow entrains local field potentials (LFPs) in the olfactory bulb (OB) and propagates respiration-linked rhythms to cortical and subcortical regions, in humans^1^^,^^2^^,^^3^ and experimental animals.^4^^,^^5^^,^^6^^,^^7^^,^^8^^,^^9^^,^^10^^,^^11^ Respiration-linked oscillations are considered to help coordinate neuronal activity and may influence cognitive and affective domains.^1^^,^^3^^,^^10^^,^^12^^,^^13^ Disrupting this sensory input, therefore, provides a plausible route through which sensory dysfunction could alter brain-wide dynamics and behavior.

Despite growing evidence linking respiration to neuronal oscillations, relatively few studies have examined how hyposmia affect oscillatory networks or their behavioral correlates. Clinically, hyposmia is common not only following viral infections—including COVID-19, where “brain fog” and attentional deficits are frequently reported^14^^,^^15^—but also across neurodegenerative and affective disorders. Olfactory impairment often precedes overt symptoms in Alzheimer’s and Parkinson’s disease^16^^,^^17^ and co-occurs with mood and cognitive disturbances in depression.^18^^,^^19^ While such observations highlight the importance of olfactory input for brain function, the mechanistic relationship between loss of smell, neural oscillations, and behavior remains poorly understood.

Nasal respiration-linked rhythms are thought to arise from the movement of air across the nasal epithelium, which activates mechanosensitive receptors expressed by olfactory sensory neurons.^20^^,^^21^ On this basis, we initially reasoned that interfering with mechanotransduction in the nasal cavity might provide a means to perturb respiration-coupled activity at its peripheral source. We therefore examined the effects of intranasal gadolinium infusion, as gadolinium is a well-established blocker of mechanosensitive ion channels in a variety of tissues.^22^^,^^23^^,^^24^

Unexpectedly, rather than producing only transient physiological effects consistent with channel blockade, intranasal gadolinium induced a pronounced but reversible disruption of the nasal epithelium, resulting in hyposmia lasting several days. Here, we exploit this effect to introduce a short-lasting, reversible rat model of hyposmia based on transient epithelial disruption. This approach avoids surgical lesions and systemic chemical exposure and permits within-subject comparisons across phases of sensory impairment and recovery. Using this model, we recorded LFPs from the OB and corticolimbic areas across wakefulness and slow-wave sleep (SWS), while simultaneously assessing anxiety-related and memory-related behaviors. In doing so, we directly test whether transient loss of peripheral olfactory input is sufficient to alter respiration-driven neural coordination and behavior, providing a mechanistic link between nasal epithelial integrity, large-scale network dynamics, and affective state.

## Results

### Gadolinium infusion to the nares produces hyposmia associated with dissociation of the nasal epithelium

**Figure 1:**
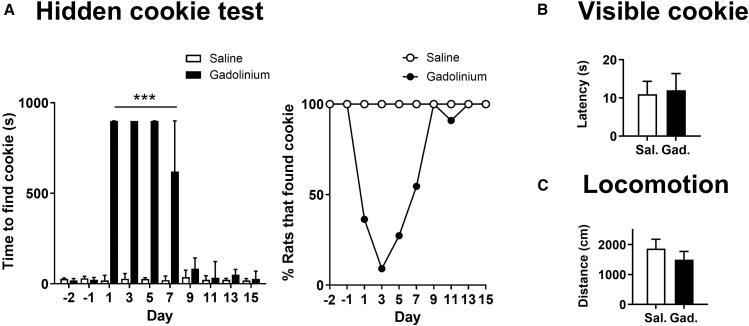
Intranasal gadolinium induces reversible hyposmia without altering motivation or locomotion (A) Latency to locate a hidden cookie two days before and up to 15 days after intranasal gadolinium chloride or saline infusion (gadolinium, *N* = 11; saline, *N* = 11). The adjacent plot shows the percentage of rats successfully locating the hidden cookie across testing days for gadolinium- and saline-infused groups. Note all rats receiving nasal infusion of saline found the hidden cookie at all time points. (B) Latency to approach and interact with a visible cookie during the motivation test performed 3 days post-infusion (gadolinium, *N* = 5; saline, *N* = 5). There was no significant difference between groups. (C) Total distance traveled in an open-field test following gadolinium or saline (gadolinium, *N* = 15; saline, *N* = 15); group differences were not statistically significant. Data are presented as mean and SEM with exception to 1A where median and interquartile ranges are shown. ∗∗∗*p* < 0.001.

We first examined whether single bilateral intranasal gadolinium (*N* = 11 rats) or saline infusion (*N* = 11 rats) affected olfactory sensitivity using the hidden cookie test. Prior to infusion, there were no differences between groups, and all rats reliably located the cookie (saline: 24.45 ± 2.29 s; gadolinium: 22.9 ± 3.48 s; Figure 1A). Following intranasal gadolinium, but not saline, we observed a marked increase in latency to find the hidden cookie with almost all gadolinium nasal-infused rats failing to find the hidden cookie within the allocated 15 min. This effect was evident as early as 1 day post-infusion (repeated-measures two-way ANOVA, group × time interaction: F(9,180) = 27.02, *p* < 0.0001; Figure 1A). Bonferroni post hoc analysis revealed significant deficits from days 1–7 (*p* < 0.001). Data were significant for the same time points using the non-parametric Friedman’s test (Friedman statistic = 60; *p* < 0.0001). The impairment peaked at day 3, when 10 of 11 gadolinium-infused rats failed to find the cookie within the allotted time. In contrast, all saline-infused rats successfully located the cookie at every time point tested. Gadolinium-induced effects on the retrieval of a hidden cookie were reversible, and by day 9 group effects no longer differed from controls. The time course of hidden cookie performance for individual rats from gadolinium and saline nasal infusion groups are shown in Figure S1.

To determine whether this deficit reflected impaired motivation or motor function rather than olfactory loss, we conducted a probe test in a separate cohort 3 days post-infusion. Gadolinium- and saline-infused rats did not differ in the latency to approach a visible cookie (*p* = 0.86, unpaired *t* test; Figure 1B). Similarly, locomotor activity measured in an open field revealed no difference in total distance traveled (*p* = 0.38, unpaired *t* test; Figure 1C). Together, these findings indicate that the impaired performance in the hidden cookie test was unlikely to result from differences in motivational or motor behaviors.

### Gadolinium infusions reduce nasal epithelial thickness and decrease OMP expression

**Figure 2:**
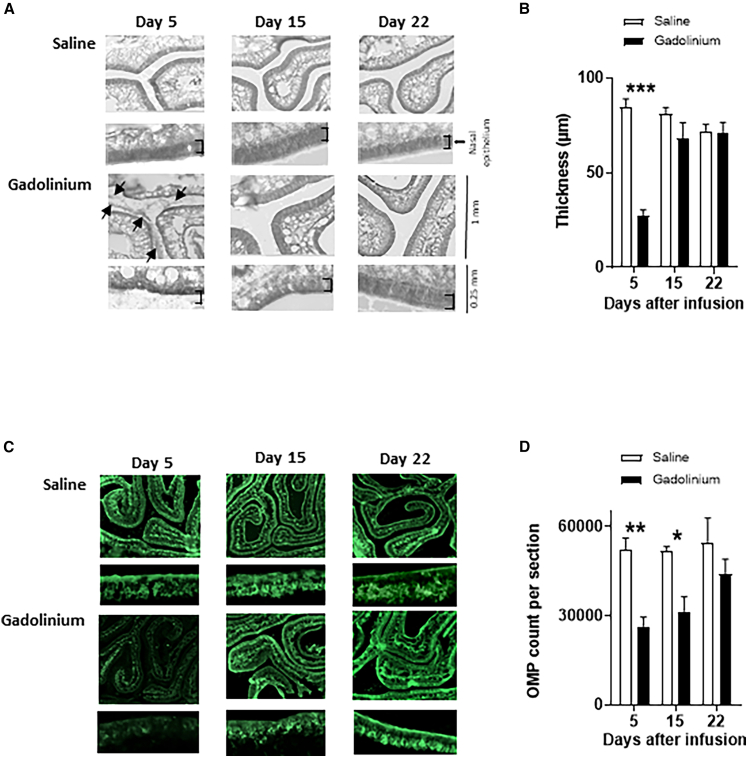
Intranasal gadolinium transiently disrupts the nasal epithelium and reduces OMP expression (A) Representative H&E-stained sections of the nasal epithelium at 5, 15, and 22 days following intranasal saline or gadolinium chloride (*N* = 4–5 rats per group per time point). Arrows indicate areas where dissociation of the nasal epithelium is visible. (B) Quantification of nasal epithelial thickness (μm) showing a significant reduction following gadolinium infusion at day 5. (C) Representative anti-OMP–stained sections of the nasal epithelium at 5, 15, and 22 days after saline or gadolinium infusion. (D) Quantification of OMP expression demonstrating reduced OMP-positive profiles following gadolinium infusion. OMP immunoreactivity is markedly reduced 5 and 15 days after gadolinium treatment (*N* = 4–5 rats per group per time point). Data are shown as mean and SEM. ∗*p* < 0.05, ∗∗*p* < 0.01, ∗∗∗*p* < 0.001.

We next examined whether the gadolinium-induced hyposmia (based on hidden cookie performance) was accompanied by histopathological changes in the olfactory epithelium. Using separate groups of rats (*N* = 4–5 per group), we assessed epithelial integrity 5, 15, and 22 days after intranasal gadolinium or saline infusion. At day 5, visual inspection of H&E-stained sections revealed that gadolinium induced widespread dissociation of the olfactory epithelium from the basement membrane, evident across both medial and lateral regions (Figure 2A). To quantify this damage, we measured epithelial thickness to generate an epithelial index, as used by others.^25^ This analysis confirmed a significant overall reduction in epithelial thickness in gadolinium-infused rats (repeated-measures two-way ANOVA, group effect: F(1,24) = 35.07, *p* < 0.0001; Figure 2B). Bonferroni post hoc tests indicated that the epithelium was significantly thinner at day 5 (*p* < 0.001) and no differences on 15 and 22. In contrast, the nasal epithelium in saline-infused animals remained stable at all time points. Dissociation of the epithelium was likely responsible for a distinct “snorting” sound frequently observed during the first few days post-infusion. Despite this structural disruption, rats showed no nasal discharge, visible inflammation, or respiratory difficulty.

Because mature olfactory sensory neurons express olfactory marker protein (OMP), we also examined OMP immunoreactivity across the same time points (Figure 2C). Gadolinium infusion significantly reduced the number of OMP-positive neurons (repeated-measures two-way ANOVA, group effect: F(1,22) = 24.1, *p* < 0.0001; Figure 2D). Bonferroni post hoc tests revealed OMP staining was reduced at day 5 (*p* < 0.01) and day 15 (*p* < 0.05), but no longer differed from controls by day 22.

### Nasal epithelial damage reduces LFP oscillations in the OB

**Figure 3:**
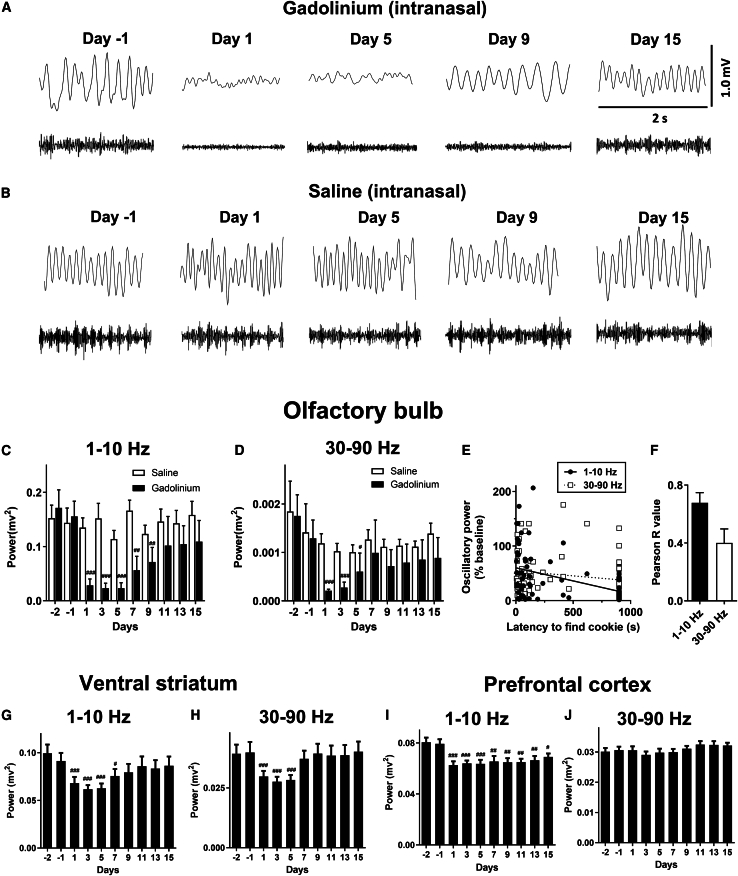
Nasal epithelial disruption reduces respiration-linked and gamma oscillations in the OB and downstream regions (A and B) Representative olfactory bulb (OB) LFPs showing respiration-linked (1–10 Hz) and gamma (30–90 Hz) activity before (day −1) and up to 15 days after intranasal gadolinium chloride or saline infusion. (C) Power of 1–10 Hz oscillations in the OB are significantly reduced following gadolinium but not saline infusion (gadolinium, *N* = 10; saline, *N* = 10). (D) Power of gamma frequency in the OB is reduced following gadolinium but not saline infusion. (E) Linear regression analyses showing the relationship between latency to locate the hidden cookie (longer latency shows greater impairment) and changes in 1–10 Hz and gamma 30–90 Hz power relative to baseline. The slope significantly differed from zero for the 1–10 Hz (solid line) band but not the gamma band (dotted line). (F) Pearson correlation coefficients (mean ± SEM) for individual rats relating hidden cookie performance to changes in oscillatory power. Significant correlations were observed in 7/10 rats for the 1–10 Hz band and 3/10 rats for the 30–90 Hz band. (G–J) In a separate group of rats (*N* = 12), we examined 1–10 Hz and 30–90 Hz oscillatory power in the ventral striatum and prefrontal cortex. Data are shown as mean and SEM. Significant differences with respect to baseline (day −2) ^#^*p* < 0.05, ^##^*p* < 0.01, ^###^*p* < 0.001 with respect to day −2.

Large-amplitude oscillations associated with nasal respiration have long been known to dominate LFP in the OB.^26^^,^^27^^,^^28^ In the present study, we focused on the 1–10 Hz band because it captured the dominant respiration-linked component of the OB LFP in our recordings across the behavioral conditions examined, and because it has been used in multiple prior LFP studies to quantify respiration-related power in rodents.^29^^,^^30^^,^^31^^,^^32^ We examined whether this rhythm was disrupted following gadolinium-induced hyposmia. Using the same rats tested in the hidden cookie task, we recorded OB LFP activity after each behavioral session (Figures 3A and 3B). One rat from each group was excluded due to a bad electrode contact which developed over the recording sessions (*N* = 10 rats for LFP analyses). Intranasal gadolinium produced a significant decrease in the power of the 1–10 Hz respiration rhythm (one-way repeated-measures ANOVA: F(9,81) = 6.56, *p* < 0.0001; Figure 3C). Time courses showing changes in 1–10 Hz power for individual rats are shown in Figure S2. Gadolinium also reduced gamma-band (30–90 Hz) power in the OB (one-way repeated-measures ANOVA: F(9,81) = 4.70, *p* < 0.0001; Figure 3D), although this effect was shorter-lived than the reduction in 1–10 Hz activity. Saline had no effect on 1–10 Hz power (F(9,81) = 1.06, *p* = 0.40) or gamma power (F(9,81) = 0.90, *p* = 0.53).

The time course of the decrease in 1–10 Hz oscillatory power closely paralleled the behavioral deficit observed in the hidden cookie test, suggesting a potential relationship between the two measures. To examine this directly, we tested whether reductions in 1–10 Hz activity were associated with impaired olfactory performance in the hidden cookie test we reported in Figure 1A. Group-level changes in 1–10 Hz power were negatively correlated with latency to locate the hidden cookie following gadolinium infusion (R = −0.43, *p* < 0.0001; Figure 3E), indicating that larger reductions in low-frequency power were associated with poorer odor-guided performance. At the individual level, Pearson correlations were significant (*p* < 0.05) in 7/10 rats for the 1–10 Hz band, compared with 3/10 rats for the 30–90 gamma Hz band (Figure 3F).

Low frequencies are known to modulate fast brain rhythms.^9^^,^^33^ In the OB baseline, condition showed clear cross-frequency coupling, with a prominent modulation cluster centered around 80 Hz (Figure S3). In contrast, the gadolinium rats exhibited reduced modulation strength across the same frequency range. Comparison of the maximum modulation index (MI) values between baseline and 3 days post-gadolinium recordings found a significant decrease in modulation following gadolinium administration (paired *t* test, *p* < 0.005).

Since nasal respiration can influence neuronal activity in other brain areas outside the OB,^1^^,^^4^^,^^11^^,^^27^^,^^34^ we implanted a separate group of rats (*N* = 12) with electrodes in the ventral striatum (VS) and prefrontal cortex (PFC) and recorded LFP oscillations every 4 days post-gadolinium/saline nares infusion. One-way repeated measures ANOVA revealed a small but significant reduction in 1–10 Hz activity (VS: F(9,89) = 10.21, and PFC: F(9,98) = 6.9, *p* < 0.0001, *p* < 0.0001 followed by Dunnett post hoc tests; Figures 3G and 3I). We also found a reduction in gamma activity in the VS (F(9,89) = 9.3, *p* < 0.0001 followed by Dunnett post hoc tests; Figure 3H) but not the PFC (F(9,98) = 0.95, *p* = 0.51; Figure 3J).

### State-dependent effects of nasal epithelial damage on OB oscillations

During wakefulness and locomotion OB dynamics are dominated by respiration-linked peripheral input, whereas during sleep top-down centrifugal cortical inputs provide a powerful drive.^35^^,^^36^ Because gadolinium infusion selectively disrupts sensory transmission from the nasal epithelium, we hypothesized that wake-related oscillations would be impaired, whereas sleep-related activity would remain largely preserved. To test this, we examined OB LFPs across two behavioral states, SWS and locomotion, in a separate cohort of rats given nasal infusion of gadolinium (*N* = 7) or saline (*N* = 5).

**Figure 4:**
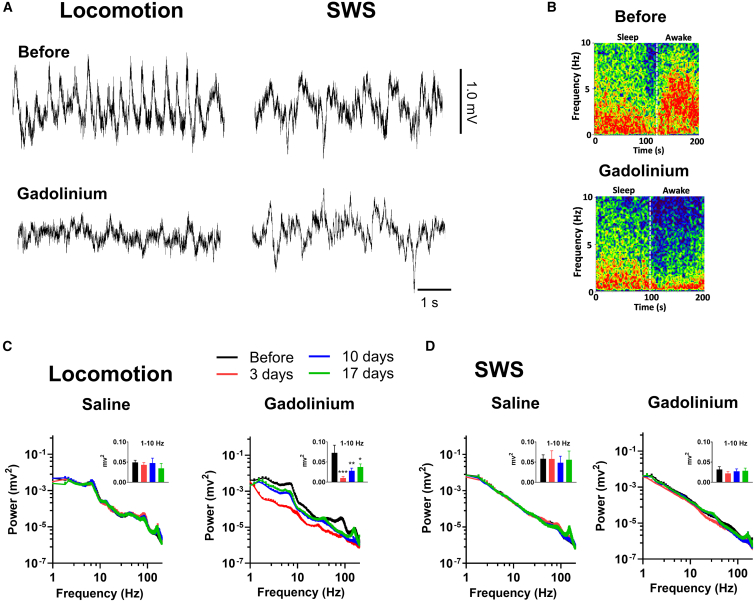
Nasal epithelial disruption selectively reduces OB oscillatory power during wakefulness but not slow-wave sleep (A) Example raw LFPs recorded from the OB during locomotion and slow-wave sleep (SWS) before and 3 days after intranasal infusion of gadolinium. Note that large amplitude deflections remained after gadolinium despite the rhythmic reduction during locomotion. (B) LFPs expressed as a spectrogram for a transition from SWS to waking showing that 1–10 Hz power was reduced in the waking state after gadolinium (same color scale for both images). (C and D) Average OB power spectra for saline (*N* = 5) and gadolinium -infused rats (*N* = 7) during locomotion and SWS. Data shown are from before (4 days pre-infusion) and 3, 10, and 17 days post-infusion. Inset bar graphs show quantification of integrated 1–10 Hz power. Data are shown as mean and SEM. ∗*p* < 0.05, ∗∗*p* < 0.01, ∗∗∗*p* < 0.001.

Representative LFPs recorded from the OB during locomotion and SWS before and 3 days after nasal infusion of gadolinium are shown in Figure 4A. Slow oscillatory activity was markedly reduced following gadolinium infusion during locomotion. However, during SWS large amplitude fluctuations in the OB remained visible. A transition from SWS to active waking before and after gadolinium infusion is shown in Figure 4B. Spectral power was used to quantify changes calculated for baseline and at 3, 10, and 17 days post-infusion in gadolinium and saline-infused during locomotion and SWS (Figures 4C and 4D). In gadolinium-infused rats, locomotion was associated with a significant reduction in OB power (group effect: F(3,18) = 5.7, *p* = 0.006; group × time interaction: F = 2.1, *p* < 0.0001). However, during SWS, low-frequency rhythms dominated the power spectrum and were not detectably altered by intranasal gadolinium (group × time interaction: F(1908,11448) = 0.96, *p* = 0.85). Analysis of integrated 1–10 Hz power confirmed this state dissociation: gadolinium significantly reduced 1–10 Hz power during locomotion (one-way ANOVA: F(3,18) = 8.1, *p* = 0.0013, followed by Dunnett’s post hoc test) but not during SWS (F(3,18) = 2.6, *p* = 0.081). We also analyzed a narrower 0.5–4 Hz band in the gadolinium group and found reduced power during locomotion post gadolinium infusions (F(3,18) = 5.6, *p* = 0.006), but not during SWS (F(3,18) = 2.08, *p* = 0.13). Nasal infusion of saline had no effect on spectral patterns at any time point (SWS: F(1917,7668) = 0.67, *p* > 0.999; locomotion: F(1917,7668) = 0.66, *p* > 0.999).

### Increased anxiety-like behavior and memory impairment following damage to the nasal epithelium

**Figure 5:**
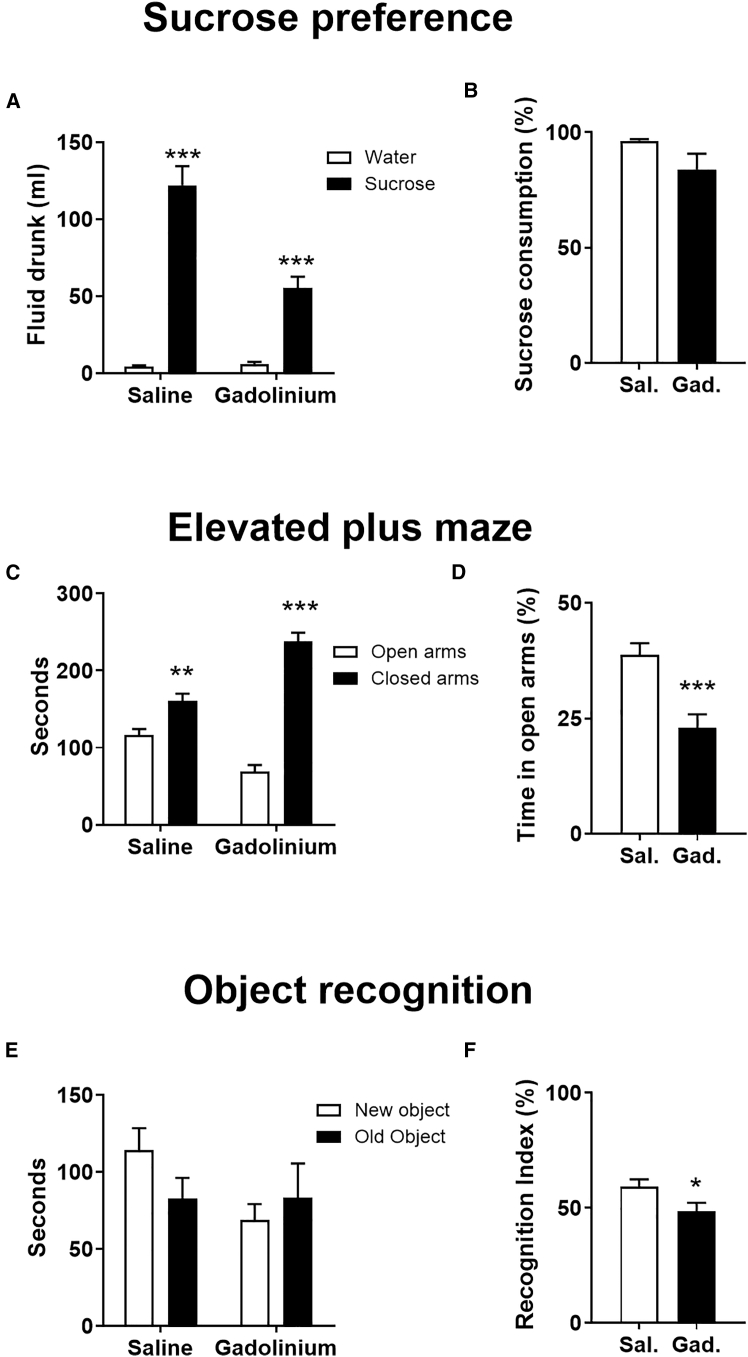
Nasal epithelial disruption produces selective behavioral alterations (A and B) Sucrose preference test showing the both saline and gadolinium infused rats have a preference for sucrose relative to water (gadolinium, *N* = 15; saline, *N* = 15, tested 2 days after nasal infusion). (C and D) Elevated plus maze showing that both saline- and gadolinium-infused rats have a preference for the closed arm, but the fear index (% time spent in open arm) is greater for the gadolinium group (gadolinium, *N* = 15; saline, *N* = 15, tested 4 days after nasal infusion). (E and F) Novel object recognition test demonstrating a small but significant reduction in recognition index following gadolinium infusion compared to saline controls (gadolinium, *N* = 10; saline, *N* = 10, tested 2 days after nasal infusion). Data are shown as mean and SEM. ∗*p* < 0.05, ∗∗*p* < 0.01, ∗∗∗*p* < 0.001.

In a separate group of rats, we carried out a battery of behavioral tests carried out between 2 and 4 days post-intranasal gadolinium or saline infusion. In the sucrose preference test, both groups of rats showed a clear preference for sucrose over tap water (F(1,28) = 21.17, *p* < 0.0001; Figure 5A), although the gadolinium group drank less fluid overall (*p* < 0.001, unpaired *t* test). Comparison of sucrose preference between the groups revealed no significant difference (unpaired *t* test, *p* = 0.08; Figure 5B).

Anxiety was assessed using the elevated plus maze and both groups of rats displayed fear avoidance with fewer entries in the open arm (F(1, 28 = 23.47, *p* < 0.0001; Figure 5C). Nasal infusion of gadolinium rats was associated a significantly greater fear index compared to saline controls spending less time in the open arm (unpaired *t* test *p* < 0.001; Figure 5D) and fewer entries to the open arm (unpaired *t* test, *p* < 0.01).

Rats underwent a novel object recognition test to investigate memory. The overall ANOVA did not reach significance (group × time interaction, F(1,18) = 3.2, *p* = 0.091; Figure 5E). However, the recognition index (N/N + F, where N is time spent interacting with novel object and F is time spent interacting with the familiar object) was lower for gadolinium rats compared to saline rats (unpaired *t* test; *p* = 0.042; Figure 5F).

## Discussion

Our findings identify a link between olfactory epithelial integrity and respiration-coupled neural dynamics. Transient dissociation of the nasal epithelium following intranasal gadolinium infusion produced a reversible impairment in odor-guided behavior and robust reductions in OB respiration-linked (1–10 Hz) and gamma (30–90 Hz) band activity, with smaller changes detected in the VS and PFC. Consistent with these network effects, we observed selective behavioral alterations, including increased anxiety-like behavior in a high-stress assay and impaired recognition memory. Together, these results suggest that transient peripheral olfactory disruption can weaken rhythmic coordination within olfactory-corticolimbic circuits and influence affective and cognitive performance.

The present study establishes a short-lasting and reversible model of hyposmia based on intranasal gadolinium infusion. All rats exhibited impaired odor-guided behavior as assessed by the hidden cookie test, with maximal deficits observed 3 days after infusion, when only 1 of 11 gadolinium-infused animals located the cookie within the allotted time compared with all saline-infused controls. Gadolinium-induced hyposmia was transient, with most animals showing recovery of odor-guided behavior by day 7 and full recovery by day 9–11.

Notably, recovery of hidden cookie performance closely paralleled the restoration of respiration-linked oscillatory activity in the OB, which may suggest a functional relationship between olfactory sensory input and OB rhythmic dynamics. Histological analyses revealed marked degeneration of the nasal epithelium and reduced OMP expression at day 5, with progressive structural recovery by day 15 and near-complete restoration by day 22. This temporal dissociation—earlier functional recovery followed by slower epithelial regeneration—is consistent with other reversible models of hyposmia^37^^,^^38^ and indicates that partial epithelial recovery is sufficient to support odor-guided behavior. In the present study, olfactory function was assessed using the hidden cookie test, a widely used assay of odor detection in rodents.^37^^,^^39^^,^^40^^,^^41^ Recovery of performance in this task demonstrates restoration of odor-guided function, however, future studies employing other tests, for example odor discrimination and detection threshold paradigms, are required to determine whether additional dimensions of olfactory perception share a similar disruption-recovery time course.

Multiple experimental models of anosmia and hyposmia have been developed, including genetic manipulations (e.g., knockout of olfactory signaling proteins such as Gαolf,^42^^,^^43^ viral infection models (e.g., SARS-CoV-2-based paradigms,^44^ olfactory bulbectomy,^45^ and chemical approaches, such as systemic 3-methylindole,^46^ methimazole,^47^ or methyl bromide gas inhalation.^48^ The gadolinium model described here is most comparable to the zinc sulfate method, one of the most widely used intranasal chemical approaches for inducing olfactory epithelial degeneration. However, zinc sulfate produces highly variable durations and severities of olfactory impairment, ranging from a few days^39^^,^^49^ to several weeks or months,^37^^,^^41^ depending on concentration, volume, and number of administrations (reviewed by McBride et al.^50^). Moreover, most zinc sulfate studies have been conducted in mice, and its effects in rats are weaker and more inconsistent.^49^^,^^51^ Thus, intranasal gadolinium offers a tractable alternative in rats, combining intranasal delivery with a reliable and time-limited disruption of olfactory function.

Overall, the intranasal gadolinium procedure was well tolerated. Rats exhibited preserved motivation (visible-cookie test) and intact locomotion (open field). This profile is broadly consistent with reports from zinc sulfate models, which typically show no major disruption of spontaneous locomotor activity,^40^^,^^52^^,^^53^^,^^54^ although reduced locomotion has been reported.^55^ Also Zhu and colleagues reported no change in total locomotion, reduced center exploration in an open field was observed.^54^ Motivation to obtain and consume a food reward also remained intact after gadolinium infusion, consistent with findings that zinc sulfate treatment does not necessarily impair appetitive drive.^39^ Apart from transient snorting or sniffing—also reported in zinc sulfate models^41^ there were no signs of overt illness, nasal discharge, or respiratory impairment.

Across all experiments, two rats died during infusion; both weighed less than 300 g and these events occurred during pilot studies using a deep anesthesia protocol with animals maintained in a supine position to promote more complete nasal perfusion. Following refinement of the procedure using lighter anesthesia and rats weighing ≥320 g, no further mortality was observed (*N* = 60 rats). The animals did not display overt signs of illness, based on visual inspection, and were largely indistinguishable from control rats (home cage environment) the following day aside from occasional snorting/sniffing. Zinc sulfate has been associated with high mortality and post infusion illness, particularly in mice and at higher concentrations or volumes.^53^^,^^55^^,^^56^

Gadolinium is known to block mechanosensitive cation channels in various tissues,^22^^,^^23^^,^^24^ which motivated its initial use in this study. However, the duration of the effects observed here far exceeded the expected timescale of channel blockade. Gadolinium is known to disrupt mitochondrial function, increase reactive oxygen species, and promote apoptosis^57^^,^^58^ which likely underlie the degeneration of the nasal epithelium we observed. Although gadolinium can accumulate in multiple organs and is potentially toxic,^59^ the parallel recovery of epithelial integrity, odor-guided behavior, and respiration-linked oscillatory activity is consistent with a localized and reversible disruption of peripheral sensory input rather than systemic toxicity.

LFPs are low frequency (typically below 300 Hz), extracellular fluctuations largely caused by postsynaptic potentials, which when synchronized produce rhythmic oscillations.^60^^,^^61^ Since the work of Lord Adrian in the 1940s, it has been known that nasal respiration can entrain oscillatory activity in the OB.^28^ These rhythms are considered to arise from stimulation of mechanoreceptors on olfactory sensory neurons across the nasal epithelium by airflow which project to the OB producing a rhythm.^20^^,^^21^^,^^62^ Work in experimental animals and humans has shown that respiratory rhythms can propagate to many brain areas and also entrain faster activity, such as gamma activity.^1^^,^^2^^,^^9^^,^^10^^,^^63^^,^^64^ Consistent with others,^7^^,^^33^^,^^64^ we observed clear coupling of gamma to slow rhythms in the OB, and this coupling was reduced after gadolinium intranasal infusion. The loss of this low-frequency scaffold that helps organize higher-frequency oscillations likely explains the accompanying reduction in gamma power observed.

Intranasal gadolinium markedly reduced 1–10 Hz activity in the OB during locomotion, however, during SWS OB activity largely resembled control rats. Others have shown that during SWS, respiration becomes slower and more irregular and can partially decouple from low-frequency OB activity, even as prominent slow oscillations persist.^26^^,^^36^^,^^65^ During sleep, top down cortical inputs can synchronize activity in the OB^35^^,^^36^ and these networks, spared by gadolinium nares infusion, likely explaining why sleep-related activity remains present.

Reductions in 1–10 Hz power in the PFC and VS during wakefulness, although smaller in magnitude than those observed in the OB, are consistent with the view that peripheral olfactory input contributes, at least in part, to the entrainment of distributed activity throughout the brain.^9^^,^^34^ However, of course slow activity in the PFC and VS is not exclusively driven by respiration; both regions possess local and network-level mechanisms capable of generating low-frequency rhythms.^66^^,^^67^^,^^68^^,^^69^^,^^70^^,^^71^ Consequently, loss of nasal respiratory input would be expected to have a proportionally weaker impact on these structures than on the OB, where respiration-linked drive is dominant. Notably, for reasons that are unclear, the PFC effect, although modest, appeared more sustained than changes observed in the OB and VS. The underlying cause is unclear; one possibility is that respiration-related coupling in the PFC is additionally driven by non-olfactory cues (e.g., temperature) that take longer to recover following epithelial disruption. Alternatively, the slower recovery profile may reflect longer-lasting neuroplastic adjustments that alter the propagation or expression of OB-driven rhythms within cortical networks.

Across many species, nasal breathing can modulate cortical gamma amplitude.^8^^,^^33^ Here, we observed intranasal gadolinium reduced gamma-band power in the OB and VS, but not in the PFC. Reductions in gamma power have also been reported after intranasal zinc sulfate in regions that include the VS and piriform cortex, and extend into dopaminergic reward circuitry such as the ventral tegmental area,^72^ indicating that peripheral olfactory disruption can influence network dynamics beyond classical olfactory structures. Mechanistically, VS gamma can be driven in part by olfactory cortical/piriform inputs^73^ and this area, in turn, receives strong excitatory input from OB output neurons.^74^ Olfactory bulbectomy—a widely used rodent model of depression^45^ is associated with reductions in gamma oscillations across limbic networks including the VS.^75^ Reinstating OB-derived gamma alleviates depression-like behavior in rodents.^75^ Together, OB-driven gamma may contribute to coordinating limbic network function, providing a plausible route by which peripheral olfactory disruption, as occur in models of hyposmia and anosmia, may alter affect-related circuitry.

In humans, olfactory impairments can significantly impact psychological well-being^76^^,^^77^ and severities of smell and taste loss have been associated with depressed mood and anxiety.^78^ However, studies examining changes in behavior in experimental animal models of olfactory impairment are relatively sparse. Here, we examined whether behavioral changes occurred in the gadolinium model of hyposmia using three tasks, sucrose preference (hedonic drive), elevated plus maze (anxiety), and object recognition (memory). We observed selective behavioral alterations in that rats exhibited increased anxiety in the elevated plus maze and also a small but significant reduction in recognition memory. Hedonic drive, assessed by sucrose preference, remained unchanged however, for reasons we are unclear gadolinium rats drank less fluid overall. Olfactory deficits have been linked to anxiety in mice.^79^^,^^80^ However, the intranasal zinc sulfate model tends to produce inconsistent effects on anxiety with increases,^54^ no change,^52^ and decreases after repeated treatments being reported in mice.^81^ Zhu and colleagues also reported reduced object recognition and spatial memory in mice.^54^ Respiration-linked rhythmic activity across distributed brain regions in humans and rodents can support affect and memory.^1^^,^^10^^,^^82^^,^^83^^,^^84^ Degeneration of the olfactory epithelium is therefore positioned to weaken this peripheral respiratory drive and reduce the ability to sustain coherent respiration-linked coordination across circuits. Although behavioral consequences of anosmia/hyposmia are likely multifactorial—including endocrine and stress-related mechanisms such as elevated corticosterone,^80^^,^^81^ we speculate that disrupted respiration-linked oscillatory dynamics contributes, at least in part, in this process.

### Limitations of the study

First, this study should be viewed as an initial proof of concept for a reversible hyposmia model. Intranasal gadolinium consistently produced a short-lived disruption of the nasal epithelium and a reversible deficit in odor-guided behavior (hidden cookie test). However, we did not perform a broader assessment of olfactory function, such as detection thresholds, odor discrimination, or odor identification across recovery. Therefore, we interpret this behavioral impairment as hyposmia rather than complete anosmia. Future work should quantify the magnitude and time course of other olfactory deficits using threshold and discrimination assays and relate these measures to epithelial recovery.

Second, although respiration-linked oscillations were robustly reduced in the OB, effects in downstream regions such as the PFC and VS were smaller in magnitude. This may reflect both attenuation of peripheral sensory drive and the presence of local and/or top-down sources of rhythmic activity in these regions. Future studies using larger-scale multi-site recordings and causal perturbations will be needed to define how olfactory-driven rhythms interact with intrinsic oscillatory mechanisms across distributed corticolimbic networks.

Third, behavioral effects were modest and selective, emerging primarily under conditions of elevated emotional demand, such as the elevated plus maze, and in recognition memory tasks, while locomotor activity and hedonic drive were preserved. Although this pattern is consistent with transient disruption of network coordination rather than global behavioral impairment, additional behavioral paradigms and longer-term assessments will be necessary to fully characterize the cognitive and affective consequences of reversible hyposmia.

Finally, all experiments were conducted in adult male rats. Given established sex differences in olfactory processing and emotional regulation,^85^^,^^86^ future studies will be needed to determine whether similar effects are observed in females and across different developmental stages.

## Resource availability

### Lead contact

Further information and requests regarding resources and analyses should be directed to and will be fulfilled by the lead contact, Mark Jeremy Hunt (m.hunt@nencki.edu.pl).

### Materials availability

This study did not generate new unique reagents.

### Data and code availability

All data reported in this article will be shared by the lead contact upon request.

This study does not report original code.

Any additional information required to reanalyze the data reported in this paper is available from the lead contact upon request.

## Acknowledgments

Financed by the National Science Centre (Poland) 10.13039/100025296OPUS 2021/41/B/NZ4/03882 and Preludium 2023/49/N/NZ4/02883. Microscopy imaging was performed at the Laboratory of Imaging Tissue Structure and Function, which serves as an imaging core facility at the 10.13039/501100014418Nencki Institute of Experimental Biology and is part of the infrastructure of the Polish Euro-BioImaging Node. Polish Node is supported by the project co-financed by the Minister of Education and Science based on contract no. 2022/WK/05 (Polish Euro-BioImaging Node “Advanced Light Microscopy Node Poland”).

## Author contributions

W.P. carried out the experimental work. W.P., A.B., and M.J.H. analyzed the data. W.P. and M.J.H. wrote the manuscript.

## Declaration of interests

The authors declare no potential conflicts of interest with respect to the research, authorship, and/or publication of this article.

## STAR★Methods

### Key resources table

**Table:** 

REAGENT or RESOURCE	SOURCE	IDENTIFIER
**Antibodies**		

Alexa Fluor 647-anti-rabbit secondary antibody	Thermo Fisher Scientific	CAT#A27040; RRID:AB_2536101
Rabbit anti-OMP	Sigma Aldrich	O7889-200UL Foreign Trade Commodity Code: 30021500
Normal goat serum	Vector Labs	CAT# S-100

**Chemicals, peptides, and recombinant proteins**		

Triton X-100	Sigma Aldrich	CAS# 9036-19-5
Fluoromount-G™ Mounting Medium, with DAPI	Thermo Fisher Scientific	CAT# 00-4959-52
Gadolinium (III) chloride hydrate	Sigma Aldrich	CAS# 19423-81-5
0.9% NaCl	Polpharma	EAN: 5903060603394
4% Paraformaldehyde	Intron	Cat# BP031
Phosphate Buffered Saline, no calcium, no magnesium	Thermo Fisher	Cat# 14190144
Isoflurane	Virbac	https://vet-uk.virbac.com/home/products/dogs/anaesthetics--analgesics/vetflurane.html
Loxicom	Norbrook	https://www.norbrook.com/us/products/loxicom-meloxicam-5-mgml-solution-for-injection/
Duracryl	Spofa Dental	https://www.spofadental.com/dental-resins/duracryl-plus-denture-base-resins
Lidocainum Hydrochloricum WZF 2 %	Polpharma	EAN: 5909990038411
Eferalgan	UPSA	https://www.upsa.com/en/produit/pain-fever/efferalgan/

**Software and algorithms**		

GraphPad Prism	GraphPad Software	https://www.graphpad.com/scientific-software/prism/
Spike2	Cambridge Electronic Design	https://ced.co.uk/products/spike2
Bonsai	Bonsai Foundation	https://bonsai-rx.org/

### Experimental model and study participant details

#### Animals

Male Wistar rats (Rattus norvegicus) weighing approximately 300 g were used in this study. A total of 95 animals were obtained from the Animal House of the Nencki Institute of Experimental Biology, Polish Academy of Sciences (Warsaw, Poland). Animals were clinically healthy and free of known pathogens at the beginning of the experiments.

Animals were housed under standard laboratory conditions at a controlled temperature of 22 ± 2°C with a 12 h light/12 h dark cycle. Rats had *ad libitum* access to standard laboratory chow and water throughout the study, unless otherwise required by specific experimental protocols (see Behavioral tests: Motivation test). Animals were housed in standard polycarbonate cages with appropriate bedding material and environmental enrichment (wooden blocks and cardboard tubes to promote natural exploratory behavior). All animals were allowed to acclimatize to the housing conditions before the start of the experiments. Animals were monitored daily for health status and general condition. The influence of sex as a biological variable was not analyzed and represents a limitation of the study.

All experiments were conducted in accordance with the European community guidelines on the Care and Use of Laboratory Animals (86/609/EEC) and approved by the 1st Local Ethics Committee for Animal Experiments in Warsaw, Poland. No new experimental cohorts or participants were recruited for this study. All experiments were conducted using the rat cohorts described in the STAR Methods section, including those used to establish the intranasal gadolinium model.

### Method details

#### Gadolinium chloride infusion

Male Wistar rats weighing over 300 g (10-12 weeks of age) were anesthetized with isoflurane and placed in a supine position. A gadolinium chloride solution was prepared by dissolving 60 mg of gadolinium chloride in 1 mL of sterile saline. While under anesthesia, 50 μL of the solution was infused into the right naris. Animals were then returned to the anesthesia chamber and maintained in a supine position for 3 minutes. This was followed by infusion of 50 μL of gadolinium chloride into the left naris, after which rats remained supine under isoflurane anesthesia for an additional 15 minutes to ensure complete perfusion of the nasal cavity.

All rats implanted with electrodes received intranasal gadolinium infusions at least 9 days post surgery, thus even rats who weighed under 300 g at time of surgery had reached the 320 g minimum weight at the time of gadolinium infusions.

#### Surgery

Male Wistar rats (280-350 g) were anesthetized using isoflurane and placed in a stereotaxic frame. During the procedure rats received butomidor (0.05 mg/kg) and carprofen (1 mg/kg) for three days post surgery. Three series of surgeries were carried out:

Series 1: OB implantation: Rats (N=22 rats, saline – N=11; gadolinium – N=11) were implanted with a pair of tungsten electrodes (125 μm, Science Products, Germany) in the OB (AP + 6.7, ML ± 0.5, DV 3–3.5 mm).

Series 2: Implantation in multiple brain regions. A separate group of rats (N=12 rats, all rats had infusions of gadolinium chloride to the nares) was implanted with a pair of tungsten electrodes (125 μm, Science Products, Germany) in the OB (AP + 6.7, ML ± 0.5, DV 3–3.5 mm), PFC (AP + 3.2, ML + 0.5, DV 3–3.5 mm) and VS (AP + 1.6, ML + 1.5, DV 7 mm). A screw posterior to the bregma was used as a reference/ground in all cases.

Series 3: Slow wave sleep study: A separate group of rats (N=12 rats, saline – N=5; gadolinium – N=7) were implanted with a pair of tungsten electrodes (125 μm, Science Products, Germany) in the OB (AP + 6.7, ML ± 0.5, DV 3–3.5 mm) and with electrocorticogram skull screw electrodes above the frontal (AP + 2.0, ML + 2.0 mm) and parietal (AP -6.0, ML + 4.0 mm) area.

#### Electrophysiological recordings

Series 1: One week after surgery, rats were placed in an arena (44 × 50 × 42 cm). LFPs were recorded through a JFET preamplifier, amplified 1000×, filtered 0.1–1000 Hz (A-M Systems, USA), and digitized at 5 kHz (Micro1401, CED, Cambridge, UK). Horizontal locomotor activity was assessed by photocell beam breaks (Columbus Instruments, USA). 20 minute baselines of electrophysiological signals were recorded 2 and 1 day prior to saline or gadolinium infusions. LFPs were recorded in freely moving rats in a separate chamber after each hidden cookie test, on days 1, 3, 5, 7, 9, 11, 13, and 15 post-infusion. Data were inspected manually and movements artefacts removed. Data were filtered for 1-10 Hz for respiration-linked and 30-90 Hz for gamma oscillations. Both frequencies were calculated from each recording site in bins of 30 second using the fast Fourier transform in Python. Due to a technical issue with the reference, the signals from two rats in the OB alone group (first series, one rat from the saline and one rat from the gadolinium group) were not used. Therefore electrophysiological data for that experiment are reported from 10 rats, however the corresponding hidden cookie data are shown from all rats (N=22 rats, saline – N= 11; gadolinium – N= 11). Rats from the series 2 experiment followed the same experiment plan as rats from the series 1 experiments, with the only 2 differences being the placement of the electrodes within the brain and a lack of the hidden cookie test prior to recordings.

#### Electrophysiological recordings of slow wave sleep

Series 3: Prior to gadolinium chloride (N=7 rats) and saline (N=5 rats) infusions, the rats were habituated to the recording cages. They were placed in them for up to 4 hours, to get accustomed to sleeping in them. A 3 hour recording of freely moving or sleeping rats was done as a pre-infusion baseline 4 days prior to infusions. Rats were recorded for up to 4 hours twice a week for 3 weeks, at 3, 7, 10, 13 and 17 days after gadolinium chloride or saline infusions.

Electrophysiological recordings were collected with Spike2 and processed in Python. The Neo library was used to process raw data files in smr format. Data were filtered using a 2nd-order Butterworth bandpass filter with cut off frequencies ranging from 0.5 to 200 Hz to remove noise while keeping the necessary frequency range. In most cases, during the recordings keyboard markers were used online to indicate sleep. LMA was confirmed as a period of increased activity recorded by the beam breaks, high intracranial EMG and a visible theta in the parietal ECoG electrode signal. Intracranial EMG activity was derived from coherent activity in pairs of electrophysiological recordings high-pass filtered >500 Hz as an estimate of muscle tone.^87^^,^^88^ The resulting signal was obtained by rectification, temporal smoothing (100 ms), and averaging across channels. Examples of frontal and parietal ECoG, iEMG, and OB LFPs during locomotion and SWS are shown in Figure S4. Epochs of 100 seconds duration were marked to capture activity during that time frame. The spectrogram was produced using a 3-second segment length, and the mean spectrogram values were determined across all epochs. The power of the SWS band (0.5-4 Hz) and 1-10 Hz was calculated by adding the spectrogram values within these ranges.

#### Phase amplitude coupling

Signals were imported from Spike2 files using the Neo I/O (Spike2IO). For each animal we loaded recordings corresponding to baseline and condition after gadolinium treatment. PAC was computed and compared between those conditions. From each file we extracted the analog channels in their recorded order and analyzed the OB signal. Signals were represented as single-channel MNE Raw Array objects (channel type: sEEG), and resampled to 1000 Hz. We then applied an FIR band-pass filter from 1 to 200 Hz using MNE. This preprocessing step was essential to remove noise and unwanted frequency components. Phase amplitude coupling was estimated with the pactools library, using the modulation-index (KL divergence based) method. For each recording we defined the phase providing frequencies from 1 to 10 Hz and the amplitude-providing frequencies from 30 to 200 Hz. To summarize coupling strength for each recording and condition we calculated the maximum modulation index within the 30-90 Hz gamma band. These values were then aggregated into a summary dataset for statistical analysis and plotting. All analyses were performed in Python using Neo, MNE-Python, pactools, NumPy, and Matplotlib.

#### Histology and tissue preparation

The nasal epithelium was examined at different time points post intranasal delivery of gadolinium or saline. Rats were deeply anesthetized with pentobarbital and perfused using phosphate buffered saline, followed by 4% paraformaldehyde at three time points: 5, 15, and 22 days after intranasal gadolinium or saline infusion (N=9 rats per timepoint, saline – N=4; gadolinium – N=5). Following perfusion, animals were decapitated and nasal epithelial and neural tissues were placed in 4% paraformaldehyde for 24 hours and then cryoprotected in 30% sucrose for an additional 72 hours. After cryoprotection, tissues were frozen on dry ice for 4 hours and subsequently stored overnight at −20°C. Samples were embedded in optimal cutting temperature (OCT) compound and sectioned using a cryostat at −20°C. The nasal epithelium was cut at 40 μm thickness, and OB sections were cut at 20 μm. Sections were mounted onto polylysine-coated microscope slides for subsequent histological and immunohistochemical processing.

Hematoxylin and eosin (H&E) staining was conducted on nasal epithelium tissue. Slides were soaked in 100%, 96% and 70% alcohol for 2 minutes in each, proceeded by 2 minutes in distilled water. They were then transferred into Harris’s Hematoxylin for 6 minutes and washed in tap water, followed by distilled water. Next, the slides were soaked in 70% and 96% alcohol, both for 2 minutes, before being transferred into Eosin Y for another 3 minutes. Finally, they were soaked in 96% and 100% alcohol for 2 minutes each, followed by a total of 6 minutes in three separate solutions of Xylene. The slides were then cover slipped using DepeX.

#### Anti-OMP immunohistochemistry

Nasal epithelium sections were outlined using a hydrophobic barrier pen and washed three times in phosphate-buffered saline (PBS), with each wash lasting 6 minutes at room temperature. Sections were then incubated for 1 hour at room temperature in blocking solution containing 5% normal goat serum and 0.3% Triton X-100 in PBS.

After blocking, sections were incubated overnight at 4°C with a rabbit anti-OMP primary antibody diluted 1:250 in blocking solution. The following day, sections were washed three times for 6 minutes in 0.3% Triton X-100 in PBS and then incubated for 2 hours at room temperature with a goat anti-rabbit secondary antibody diluted 1:500 in 0.3% Triton X-100 in PBS. Sections were subsequently washed three times in PBS for 6 minutes each, counterstained with DAPI, cover slipped, and sealed. Images were acquired using an Olympus VS110 slide-scanning microscope.

#### Quantification of OMP-positive cells and epithelial thickness

OMP-positive cells were quantified using QuPath software. Digital images were analyzed with the positive cell detection algorithm to determine the number of OMP-positive profiles in each section. One representative section spanning the entire nasal cavity was analyzed per rat.

Nasal epithelial thickness was quantified using DLT Cam Viewer software. For each rat, three nasal cavity fragments were stained with H&E. Images were acquired using an Olympus VS110 microscope at 4.5× magnification. Epithelial thickness was measured in pixels at 30 distinct locations across the nasal cavity and converted to micrometers for statistical analysis.

#### Behavioral tests

##### Hidden cookie test

The hidden cookie test is a variation of the buried food test, which is widely used to observe olfaction ability in rodents.^89^^,^^90^^,^^91^^,^^92^^,^^93^^,^^94^^,^^95^ Twenty-two adult male Wistar rats were used in this experiment. Animals were trained for two weeks to locate a Chocapic™ cookie buried under 3 cm of saw dust bedding in a cage resembling their home environment (59.5 x 38 x 20 cm). The cookie was hidden in a different spot under the bedding during each trial, but in the same place for all rats during a single trial. The animals were placed at the middle of the entrance of the cage facing forwards and were given up to 15 minutes to locate the hidden cookie. The time to find the cookie was defined as the time from entrance into the cage to the time of holding the cookie in the paws by the animals. The time was recorded using a stopwatch. Bedding was changed in between each rats’ trial in order to remove all scents. Once all rats consistently located the cookie within 60 seconds, a baseline test was followed by intranasal infusion of either gadolinium (N=11 rats) or saline (N=11 rats). Rats were tested every other day on days 1, 3, 5, 7, 9, 11, 13, and 15 post-infusion. During each session, animals were given 15 minutes to locate the cookie. Failure to locate the cookie within this time was scored as a failed trial and written down as the maximum 15 minutes.

These were the same rats which were also implanted with electrodes in the OB and recorded in a separate chamber after the hidden cookie test. This permitted us to examine the relationship between deficits in finding the hidden cookie and changes in LFP oscillations.

##### Motivation test

Rats were food deprived for 18 hours prior to testing. Three days after gadolinium infusion, rats (N=10 rats, saline – N=5; gadolinium – N=5) were placed individually at the entrance to the testing cage. A single Chocapic® cookie was placed randomly close to one of the corners of the cage. The latency to interact with the cookie was recorded and used as an index of motivation to retrieve the food reward.

##### Open field test

The open field test was conducted three days after saline (N=15 rats) or gadolinium (N=15 rats) intranasal infusion. Each rat was placed individually in the center of a 100 × 100 × 40 cm arena and allowed to freely explore for 10 minutes. The arena was empty and cleaned between animals. Testing was conducted under 150 lux illumination. Locomotor activity was recorded using a camera positioned above the arena. After testing, rats were returned to their home cages.

##### Sucrose preference test

Rats (N=30 rats, saline – N=15; gadolinium – N=15) were habituated to sucrose solution for two days prior to intranasal saline or gadolinium infusion. During habituation, the standard water bottle in each home cage was replaced with a 2.5% sucrose solution for 2 hours, after which it was returned to water.

The sucrose preference test was conducted overnight two days after intranasal infusion. Rats were individually housed in cages containing two pre-weighed bottles: one with water and one with 1% sucrose solution. Animals were allowed 18 hours of free access to both bottles. After the test, both bottles were reweighed to quantify liquid consumption. Rats were then returned to their home cages with access to water only.

##### Novel object (memory) test

The novel object recognition task consisted of three stages.

Habituation: Three days before intranasal infusion, rats (N=30 rats, saline – N=15; gadolinium – N=15) were placed individually into a 100 × 100 × 40 cm arena with no objects present and allowed to explore for 20 minutes. Animals were then returned to their home cages, and the arena was cleaned before the next animal. This habituation session was repeated 4 hours later.

Familiarization: Two days before gadolinium infusion, each rat was returned to the same arena containing two identical objects placed 10 cm apart at the center of the cage. Objects were cylindrical, white, and heavy enough to prevent displacement by the rat. Animals were allowed to interact with the objects for 20 minutes before being returned to their home cages. The arena was cleaned between animals.

Test: Two days after saline (N=15 rats) or gadolinium (N=15 rats) infusion, rats were placed back into the arena containing one familiar object and one novel object. The novel object consisted of a transparent cube with a sphere on top and was similarly weighted to prevent displacement. Objects were placed 10 cm apart. Rats were allowed 20 minutes of exploration. All stages were conducted under 150 lux illumination, and behavior was recorded using a camera positioned adjacent to the light source.

##### Elevated plus maze test

The elevated plus maze test was performed four days after saline (N=15 rats) or gadolinium (N=15 rats) intranasal infusion. The maze consisted of four arms (50 × 10 cm), two open and two enclosed by 30 cm-high walls, and was elevated 50 cm above the floor. Each rat was placed in the center of the maze and allowed to explore for 5 minutes under 150 lux illumination. Behavior was recorded using a camera positioned near the light source. After testing, rats were returned to their home cages, and the maze was cleaned before the next animal.

Data from the open field test, elevated plus maze and novel object recognition test was analyzed using the Bonsai program. Every mp4 file was cropped only to the area of interest (the entire open field for the open field test, the open and closed arms for the elevated plus maze, and an area of 5 cm around the objects for the novel object recognition test). The threshold to analyze the data was established manually, to best use the centroid function. Functions for ROI activity and ROI activity detection were used, with the numbers being saved in an excel file. The total number of seconds was calculated for each excel file with a Python script.

### Quantification and statistical analysis

Statistical analyses were performed using GraphPad Prism (version 11), Python (SciPy, NumPy, pactools), Bonsai, and QuPath software. Spectral power analyses were performed using custom Python scripts (SciPy), and phase–amplitude coupling (PAC) analysis was performed using the Python pactools package. Quantification of OMP-positive cells was performed using QuPath software. LFP signals were imported, processed, and visually inspected using Spike2 software (Cambridge Electronic Design, UK). Further signal processing and quantitative analyses were performed using custom Python scripts.

Time-course data were analyzed using repeated-measures ANOVA followed where appropriate by Bonferroni or Dunnett post hoc tests to compare gadolinium-treated animals with saline controls or post-infusion values with pre-infusion baseline, respectively. Non-parametric repeated measures data were analyzed using Friedman’s test. Comparisons between two independent groups were performed using unpaired Student’s t-tests, while paired t-tests were used for within-subject comparisons where appropriate. Pearson correlation coefficients were calculated to assess relationships between respiration-linked oscillatory activity, gamma oscillatory power and latency to locate the hidden cookie.

Behavioral data from the open field test, elevated plus maze, and novel object recognition test were analyzed using Bonsai software.

Data are presented as mean ± SEM unless otherwise stated. In Figure 1A, data are presented as median with interquartile range. The value of N represents the number of animals. Statistical significance is indicated as p < 0.05.
