# Supplementary material for: A reversible rat model of hyposmia affects respiration-linked brain oscillations and behavior
Source: iScience. 2026 Apr 15;29(5):115760. doi: 10.1016/j.isci.2026.115760 (PMC13157005; doi:10.1016/j.isci.2026.115760)

iScience, Volume *29*

Supplemental information

A reversible rat model of hyposmia affects respiration-linked brain oscillations and behavior

Wiktoria Podolecka, Aleksandra Bramorska, and Mark Jeremy Hunt

Figure S1. Latency to find the hidden cookie for all rats from the gadolinium and saline nasal infusion groups. Data show 2 days before (-2, -1) and up to 15 days post nasal infusion. The maximum length of time given for each rat to find the hidden cookie was 900 seconds. Note that all gadolinium rats showed impairment post nasal infusion whereas all saline rats quickly found the hidden cookie at all time points.

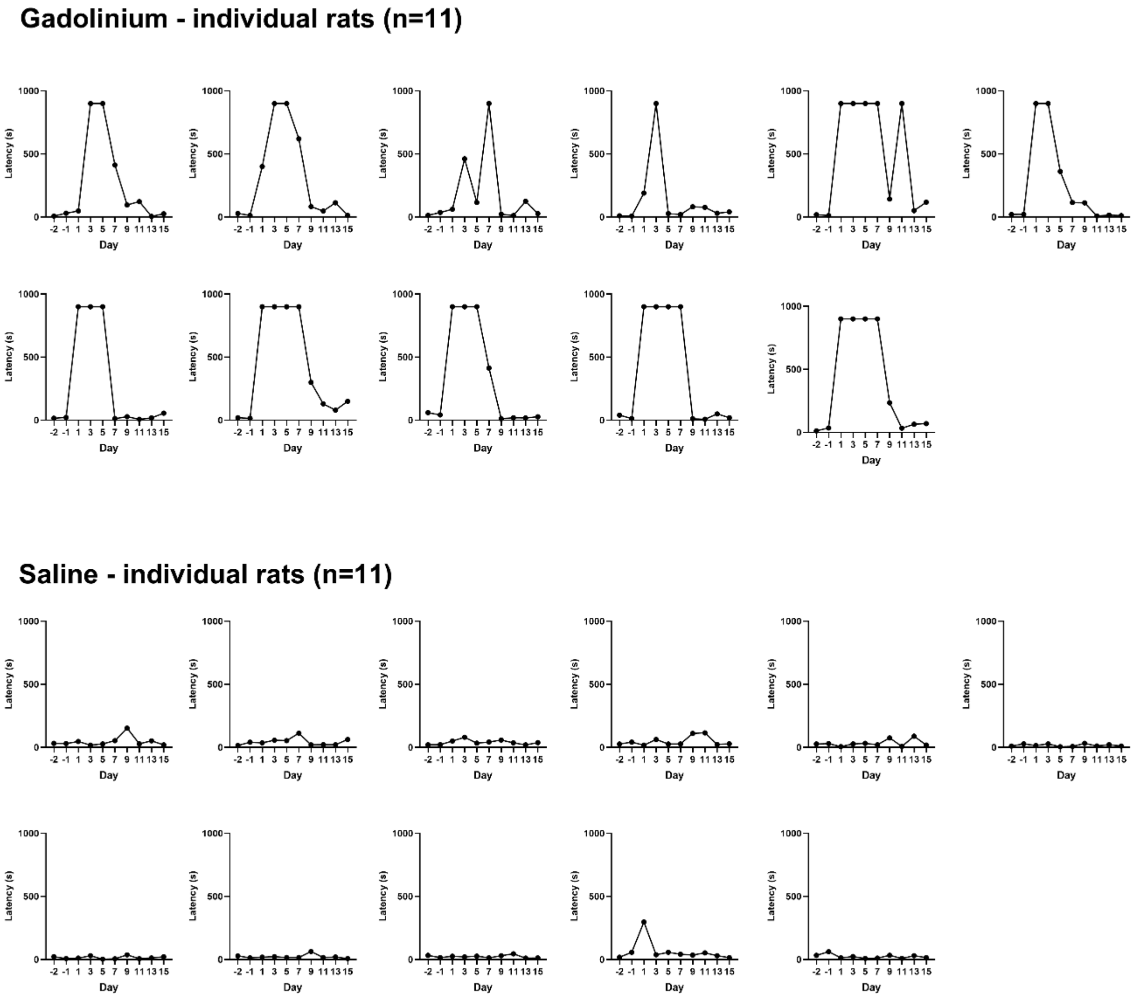

Figure S2. 1-10 Hz oscillation power in the OB before (-2, -1) and up to 15 days post intranasal infusion of gadolinium or saline.

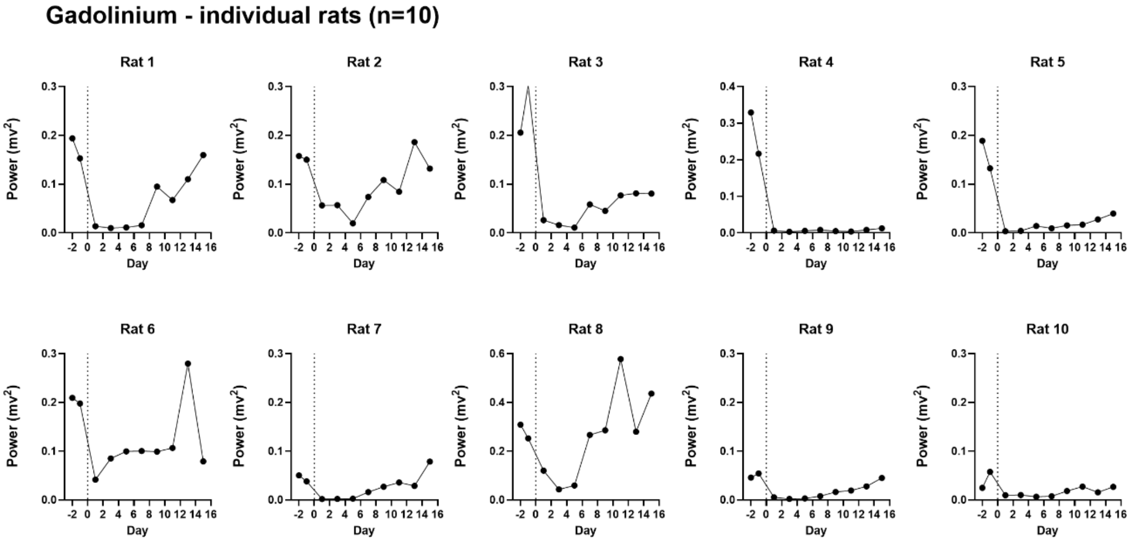

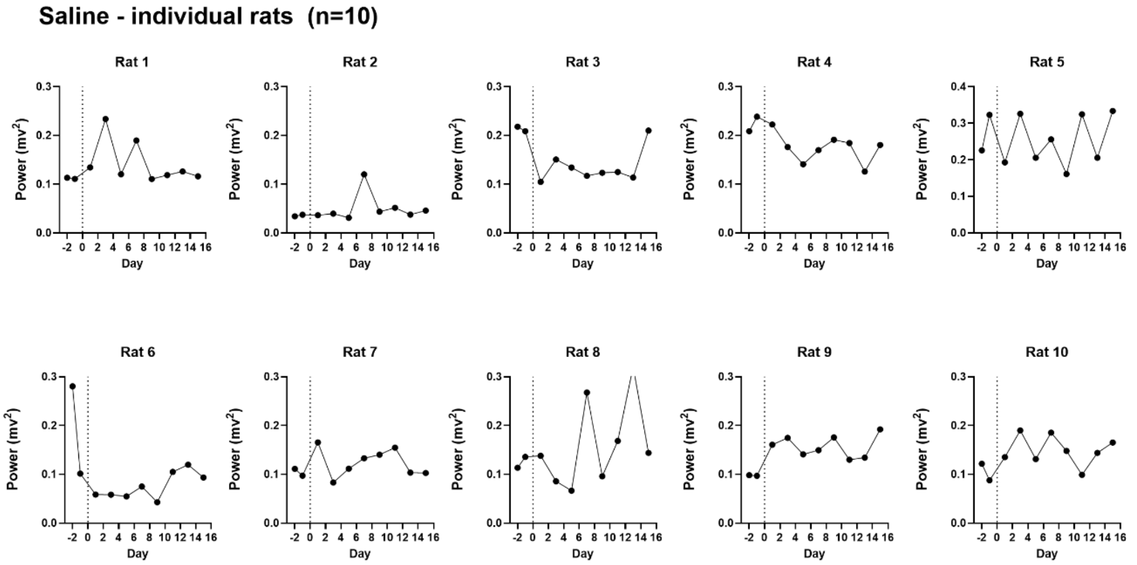

Figure S3. Mean comodulograms before and 3 days after intranasal infusion of gadolinium. Right shows the maximal modulation index for individual rats.

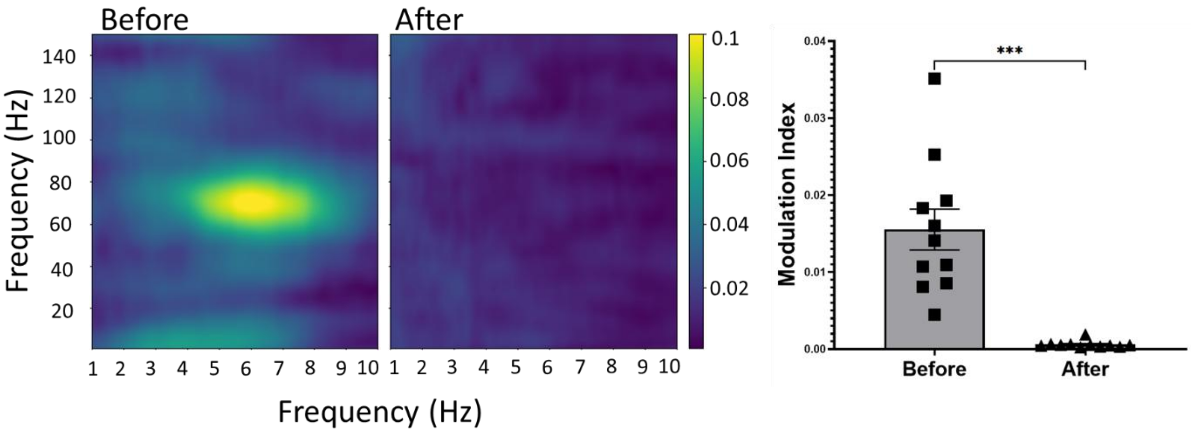

Figure S4. Characteristic electrophysiological signals during locomotion and slow wave sleep before and after gadolinium intranasal infusion. The recordings are from the same rat and show intracranial EMG, parietal and frontal ECoGs and corresponding LFP from the OB. Note that after gadolinium field potentials recorded in the OB are reduced (before vs. after), but during sleep the field potentials from the OB appear similar.

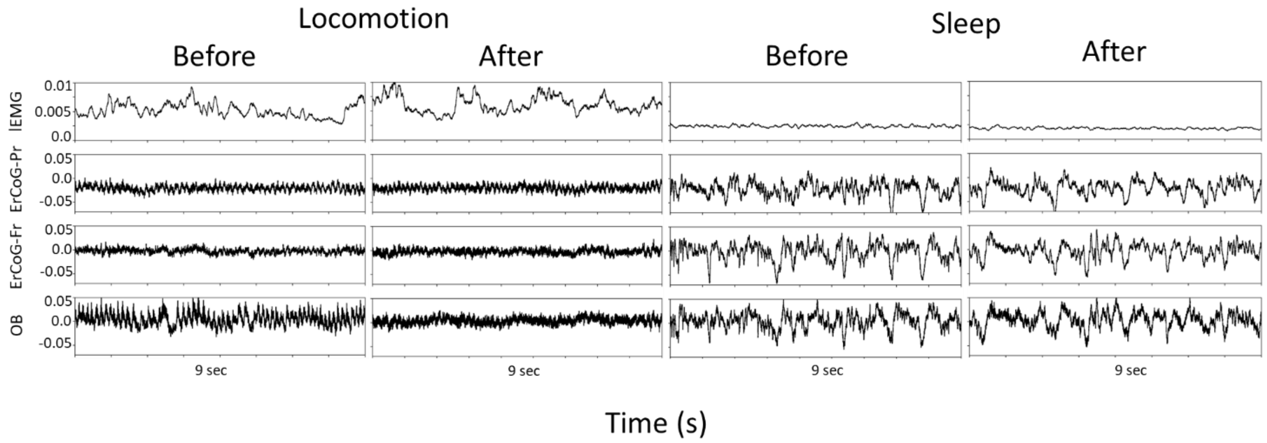

Supplement: Document S1. Figures S1–S4 [file mmc1.pdf]
